# Treatment Courses of Patients Newly Diagnosed with Multiple Sclerosis in 2012–2018

**DOI:** 10.3390/jcm12020595

**Published:** 2023-01-11

**Authors:** Jussi O. T. Sipilä

**Affiliations:** 1 Department of Neurology, North Karelia Central Hospital, Siun Sote, 80210 Joensuu, Finland; jussi.sipila@utu.fi; 2 Clinical Neurosciences, University of Turku, 50520 Turku, Finland

**Keywords:** adverse effects, disease modifying therapies, escalation therapy, immunomodulation, multiple sclerosis, pharmaceutical preparations, therapeutics

## Abstract

Treatment options for multiple sclerosis (MS) are now numerous, but it is unclear which Disease-Modifying Treatment (DMT) is the optimal choice for a given patient. Treatment switches are common, both because of side effects and because of lack of efficacy. There are few data available on the treatment courses of patients newly diagnosed with MS in the current DMT era. All patients newly diagnosed with MS in 2012–2018 at North Karelia Central Hospital were identified (N = 55), and those with complete follow-up data available (N = 43) were included. The minimum follow-up from diagnosis was 44 months with a maximum of 9 years. Seven patients (16%) had no DMT at any time during the follow-up. Treatment was most often initiated with interferon or glatiramer acetate (69%), but 72% of these treatments were discontinued. After cladribine, teriflunomide and fingolimod showed the best treatment persistence. Patients who experienced their first MS symptoms at ≥40 years of age all continued with their initial treatment category until the end of the follow-up. In a third of the patients who had received a DMT, at the end of the follow-up, the treatment had been escalated to fingolimod, cladribine or natalizumab. Only 13 patients (28%) continued with their initial DMT until the end of the follow-up.

## 1. Introduction

The past decade has multiplied our armamentarium of immunomodulatory treatment options against the inflammatory component of multiple sclerosis (MS). Nevertheless, there is still no clarity about which Disease-Modifying Treatment (DMT) is optimal for an individual patient [1,2,3]. Therefore, the choices reflect a trial-and-error approach, with DMT discontinuations and switches being common phenomena [4,5,6]. Information is limited concerning the reasons and time trajectories in clinical practice, especially considering newly diagnosed patients to whom the most recent DMTs have been available.

There is also the problem of treating MS with its increasingly complex options in more remote practices where neuroimmunological expertise is not readily available. Finland is a high-risk area for MS, but within the country, there are marked differences, with MS being apparently the most uncommon in the easternmost part, North Karelia [7]. This is a quite rural region with a rather low population density and long distances. The hospital district of North Karelia does not participate in the national MS registry [8], which recently showed that the use of natalizumab, alemtuzumab, ocrelizumab or rituximab as the first DMT was associated with a reduced risk of 5-year disability progression and relapse compared to other DMTs. The study also reported that 12.4% of the patients who had started with another DMT eventually escalated to natalizumab, alemtuzumab, rituximab or ocrelizumab at a median of 2.4 years [9]. Recent Finnish data also showed an increase in DMT switches after new oral therapies became available [10]. However, in contrast to Denmark and Sweden [4], there are no data available on individual treatment courses or reasons for discontinuing or switching DMTs in Finnish MS patients in the current treatment era.

## 2. Materials and Methods

All persons treated for MS in North Karelia Central Hospital (NKCH) in 2012–2018 were identified from hospital registries and clinical data obtained from electronic health records and were last updated on 6–7 August 2022. NKCH is the only hospital in the hospital district of North Karelia (total population of 163,000–169,000 during the study period) and therefore the only health care unit that provides MS diagnostics in the area. The follow-up of persons receiving DMTs is also provided only by NKCH in the area, although persons living in some municipalities in the border areas of the large region may be cared for by hospitals in neighboring hospital districts because they are nearer. Only persons with complete data from diagnosis until the end of the follow-up or death at NKCH were included. All interferon and glatiramer acetate treatments were analyzed as the category “Platform treatments”. All patients had at least 44 months of follow-up after the MS diagnosis.

During the study period, there were 6–9 neurologists working in the NKCH department of neurology at any given time. All were general neurologists with no special training in neuroimmunology. MS diagnoses were made and treatments were chosen following the national Current Care guidelines [11] and using shared decision making with the patient, who was given information on all available first-line therapies at the time of diagnosis and, after time to consider, chose the most appropriate one with the neurologist. In highly active disease cases, treatment decisions were also discussed between the neurologists in weekly consultation meetings, and, in the most challenging cases, Kuopio and Tampere university hospitals were also consulted. It should also be acknowledged that reimbursement protocols and decisions also influenced the treatment decisions [12].

This study was approved by the registry holder, Siun Sote (1328/13.00.01.02/2020). This was a retrospective register study, and the participants were not contacted; thus, no ethical board review or informed consent was required. The legal basis for processing personal data is public interest and scientific research (EU General Data Protection Regulation 2016/679, Article 6(1)(e) and Article 9(2)(j); Data Protection Act, Section 4 and Section 6).

Shapiro–Wilk and Kolmogorov–Smirnov tests were used to assess the distribution of continuous variables, and Student’s *t*-test, the Mann–Whitney U test or the independent-samples Kruskal–Wallis test was then used as appropriate. Cross-tabulation and the Chi-squared test were used to assess differences between groups when two variables were involved. Statistical significance was inferred at a *p*-value < 0.05. Analyses were conducted using SPSS Statistics, version 27.

## 3. Results

The search identified 55 persons who had been diagnosed with MS in NKCH during the study period. Complete follow-up data were available for 43 RRMS patients (7 were excluded because of primary progressive MS, and 5 were excluded because they had moved elsewhere during the follow-up), of whom 24 (56%) were women (Appendix A). The disease was considered highly active [11] in 17 patients (40%) at the time of diagnosis. One patient had died at >70 years of age in NKCH because of an acute myocardial infarction 7 years after the MS diagnosis. Complete follow-up data until then were available, and the patient was included in the analyses.

At the end of the follow-up, 35 patients (81%) were in remission and 2 (5%) were clearly not, whereas 6 (14%) were deemed unclassifiable because of a recent treatment switch (3 of these patients had stopped their previous treatment because of side effects and had no evidence of clinical disease activity at the time of data retrieval, 1 had just reinitiated self-stopped fingolimod because of clinical worsening, and 2 had possible but uncertain disability accrual without other signs of disease activity). Of those in remission, there were recent MRI data available for 19 (54%) to corroborate this.

No DMT had been started at any point for seven persons (16%). Nearly all of these patients had a mild course of the disease, with some experiencing symptoms once in a decade, and it was the patient’s wish that no treatment be initiated. Additionally, in two cases, the formal DMT reimbursement criteria in Finland were not met. One patient already appeared to be in a secondary progressive stage at the time of diagnosis at EDSS > 6 and chose not to initiate treatment.

**Figure 1:**
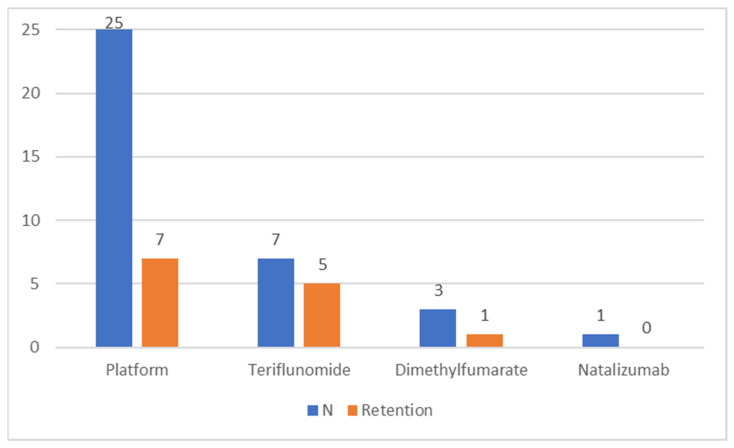
The number (N) of treatment initiations per medication/category and the number of these that remained in use until the end of the follow-up (retention).

In 69% of cases in which a DMT was initiated, the first compound was a platform drug, with teriflunomide coming second (Figure 1). In addition, one person discontinued the initiated interferon treatment within 5 months because of side effects and has remained clinically stable since (9 years). In 28% of cases in which the initial DMT was a platform drug, the patient also finished the follow-up with one, whereas 71% of the persons whose treatment started with teriflunomide also finished the follow-up with it (Figure 1). The only natalizumab initiation was planned as a temporary 4-month course when it was started.

The patients who began and finished the follow-up with a platform DMT were no different in age at first symptoms (*p* = 0.069), age at MS diagnosis (*p* = 0.11) or sex distribution (*p* = 0.78) when compared to those who started with a platform drug but finished the follow-up with another kind of DMT. In the cases when the initial platform therapy was switched to another DMT category because of inefficiency, this occurred after a mean exposure of 20.4 months (standard deviation 9.1 months, range 7–31 months), whereas in cases when the initial platform treatment was discontinued or switched because of adverse effects, this occurred after a median of 6.5 months (interquartile range 25 months, range 1–81 months). Considering all initial DMTs, a switch to another category occurred at a mean of 22.3 months (standard deviation 10.1 months, range 7–35 months) when due to inefficiency and after a median of 7 months (interquartile range 34 months, range 1–81 months) when due to adverse effects.

**Figure 2:**
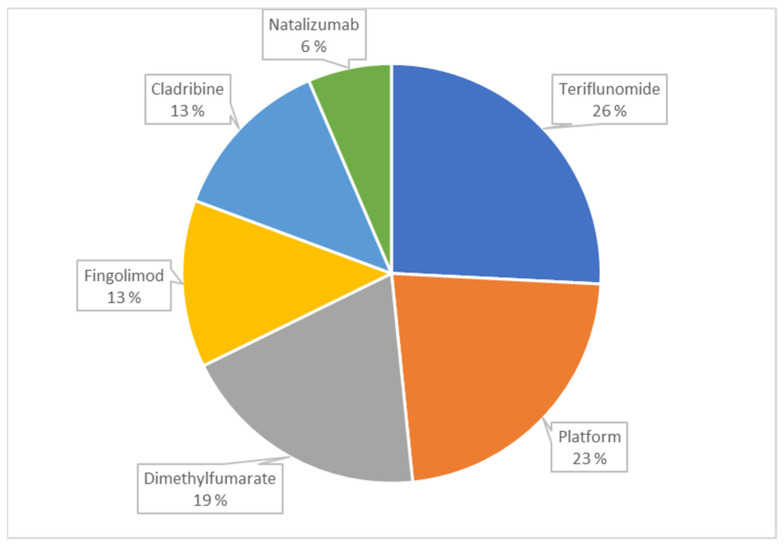
The proportions of different Disease-Modifying Treatments among the 30 patients who received one at the end of follow-up.

Excluding platform therapies, the most often used DMT was dimethyl fumarate, followed by teriflunomide, which also had the second highest persistence rate after cladribine at the end of the follow-up, whereas dimethyl fumarate had the lowest (Table 1). Among the 30 patients who received a DMT, at the end of the follow-up, teriflunomide was the most common, followed by platform therapies and dimethyl fumarate (Figure 2).

Platform therapy had been switched to another kind of DMT ten times because of side effects, nine times because of a lack of efficacy, once because of both and once for other reasons. All teriflunomide discontinuations were due to adverse effects. Fingolimod was discontinued twice due to pregnancy planning and once due to macular edema. Natalizumab was switched twice because the treatment had been planned as temporary to begin with. Only one cladribine treatment was in its third year or longer.

No patients who had had their first MS symptoms at age ≥40 needed to change their DMT category during the follow-up (four initiated with a platform drug, two with teriflunomide, one with dimethyl fumarate and two initiated with no DMT). This was not observed when analyzing age at MS diagnosis.

## 4. Discussion

This single-center observational study following 43 patients with newly diagnosed MS for at least 44 months showed that, in Finnish peripheral practice, DMTs are often switched both because of adverse effects and because of inefficacy. Almost three-quarters of the patients who received platform therapy as the first DMT finished the follow-up with a DMT of another category. The majority of patients for whom dimethyl fumarate was prescribed at some stage ended up discontinuing the drug, whereas teriflunomide and fingolimod showed higher treatment persistence. Of the patients who had a very active disease at diagnosis, the majority persisted with non-high-efficacy DMTs, even platform treatments, until the end of the follow-up, but 41% ended the period using fingolimod, natalizumab or cladribine. No patients who had their first MS symptoms at ≥40 years of age needed to switch from their initial treatment to another DMT category during the follow-up.

In the current study, only 36% of the patients for whom a DMT was prescribed continued with a similar compound until the end of the follow-up. The search for an optimal DMT is often long and arduous for both the patient and the health care system. This results from the great variability in MS disease courses and our limited ability to predict them, as well as the uncertainty about how an individual patient will fare with a given DMT. As a general rule, “lower-efficacy therapies with a known and relatively safe risk profile are selected for initial treatment. If—despite sufficiently long and regular treatment—disease activity persists/recurs, treatment is escalated to a more potent therapy option”; on the other hand, “treatment can be initiated with a high-efficacy DMT already at the time of diagnosis, for example, with alemtuzumab, cladribine, natalizumab, ocrelizumab, ofatumumab, or S1P modulators (fingolimod, ozanimod, ponesimod)” [1]. However, the clinician must always consider the individual characteristics of the patient and, preferably, share decision making. Expertise in neuroimmunology would greatly help this, but, when unavailable, data from real-world treatment courses are needed. These can also help in designing health system interventions that decrease the burden on both the patient and the health care system in finding the appropriate DMT while simultaneously providing better disease control [13].

Discontinuing platform therapies has become increasingly common [4,10]. Previous Finnish data also show better overall treatment persistence with oral therapies compared to injectables [10]. The current data also show a very high platform therapy discontinuation rate, suggesting that these DMTs usually fail the current standards of treatment. Teriflunomide, fingolimod, dimethyl fumarate, cladribine and even natalizumab all showed considerably higher treatment persistence and should be considered practically superior to platform therapies. However, the current data nevertheless suggest that platform therapies may be a reasonable option for patients who have their first MS symptoms at ≥40 years of age, especially considering the increased variety of injectable options available, their low interaction potential and little or absent need for laboratory monitoring. In patients with an earlier symptom onset, the current data suggest that teriflunomide might be considered the first option by the general neurologist and his/her patient. The good efficacy and tolerability of teriflunomide are also shown by a previous trial and real-world data [14].

Treatment should be started based on the careful consideration of poor prognostic markers (gadolinium-enhancing lesions, spinal cord and brainstem lesions, high lesion load and clinical consequences of the first attack). The initial treatment was a high-efficacy DMT in only one patient (natalizumab), whereas in a third of the patients who received a DMT, at the end of the follow-up, it was fingolimod, natalizumab or cladribine. Even though a surprisingly high proportion of patients who had very active MS at the time of diagnosis had their disease controlled with dimethyl fumarate, teriflunomide or even platform therapies, 41% of the patients with an initially very active disease ended up with fingolimod, natalizumab or cladribine. It therefore appears that, in this patient group, these DMTs should be considered as the first choice even by general neurologists, especially as an escalation strategy seems to be inferior to initially using high-efficacy therapy in the real world, and the time window in which treatment has the potential to impact the disease course of MS may be limited [1,3,9]. However, since non-high-efficacy and even platform therapies resulted in even longer than nine-year remissions in some highly active cases in these data, these therapeutic options should not be dismissed out of hand. Nevertheless, when chosen, patients should be closely monitored for signs of MS activity and the need for therapeutic escalation. Considering previous and current data, insufficient efficacy should usually become evident within the first two years [9]. In addition to fingolimod, natalizumab or cladribine, the general neurologist should consider both ocrelizumab (used so far almost exclusively for primary progressive MS in NKCH, with meager results) and rituximab (currently not used for MS in NKCH) as high-efficacy options [15,16,17,18,19,20,21].

The current data are of limited generalizability. The number of patients was low, and the data were obtained retrospectively. Since both teriflunomide and dimethyl fumarate became available in Finland only during the study period, they had been less often used as an initial DMT than platform therapies, and exposures to them were shorter. Nevertheless, with a minimum patient follow-up time of 44 months, the current data also offer valuable information on these compounds in real-world practice in non-academic centers without neurologists specialized in MS.

In conclusion, DMT switches were common, especially with platform therapies, although they seemed to work well for patients with an MS onset at >40 years of age. Teriflunomide and fingolimod had the highest treatment persistence. Although high-efficacy therapies were not needed for all patients with an initially highly active disease, these DMTs should be considered more often for these patients. No evidence of disease activity (NEDA) should, at least clinically and preferably also radiologically, be the goal.

## Figures and Tables

**Table 1:** The number of patients in whom a Disease-Modifying Treatment was initiated and the proportion of these retained at the end of follow-up, along with the number of discontinuations and their reasons.

	Initiated	Discontinued	Reasons for Discontinuation				In Use	Persistence %
			A	B	C	D		
**DMF**	14	8	3	4		1	6	43%
**TFM**	12	5		5				58%
**FNG**	7	3		1		2	4	57%
**NTZ**	4	2				2	2	50%
**CLA**	4	0					4	100%

Reasons for discontinuation: A = lack of efficiency; B = side effects; C = lack of efficiency and side effects; D = other. CLA, cladribine; DMF, dimethyl fumarate; DMT, Disease-Modifying Treatment; FNG, fingolimod; NTZ, natalizumab; TFM, teriflunomide.

## Data Availability

The data are subject to third-party restrictions. Access may be applied for through Siun Sote.

## Appendix A. Individual Patient Characteristics

**Table:**

	Age at dg	Age at 1. s	Disease Activity	1. DMT	Exposure	Reason for Switch	2. DMT	Exposure	Reason for Switch	3. DMT	Exposure	Reason for Switch	4. DMT	Exposure	Reason for Switch	5. DMT	Exposure	Remission	MRI Control
**M**	25–29	20–24	1	INFb-1a	5		DMF	48	D	DMF	6							1	1
**F**	46–50	20–24	2	INFb-1a	31	A	FNG	44	D	FNG	4							9	0
**M**	20–24	20–24	2	INFb-1a	28	A	DMF	6	A	NTZ	76							1	1
**F**	56–60	55–59	1	GA	80													1	1
**F**	40–44	35–39	0															1	1
**M**	45–49	45–49	2	INFb-1a	72	B	pINFb-1a	53										1	1
**F**	40–44	40–44	1															1	0
**F**	35–39	35–39	1	GA	30	A	DMF	48	B	TFM	2	B	pINFb-1a	2	D	CLA	12	1	0
**F**	25–29	25–29	2	INFb-1a	6	B	DMF	25	A	CLA	36							9	1
**M**	30–34	30–34	1	INFb-1a	48	D	pINFb-1a	21	A	DMF	44							1	1
**M**	55–59	35–39	1	INFb-1a	81	B	TFM	2										9	0
**M**	25–29	20–24	2	NTZ	6	D	INFb-1a	54	A	FNG	61							1	1
**F**	40–44	20–24	1															1	0
**M**	25–29	25–29	0	INFb-1a	5	B												1	0
**M**	30–34	30–34	2															1	0
**M**	35–39	25–29	2	INFb-1a	1	B	GA	10	B	DMF	83							1	0
**M**	50–54	25–29	1	INFb-1a	14	A	FNG	3	B	NTZ	4	D	DMF	85				1	0
**F**	25–29	25–29	1	TFM	39	B	pINFb-1a	5	B	DMF	29							1	1
**M**	20–24	20–24	2	INFb-1a	19	A	NTZ	92										1	1
**F**	60–64	50–54	0															0	1
**F**	30–34	30–34	2	INFb-1a	7	A	FNG	31	D	TFM	5	B						1	1
**F**	50–54	35–39	1															0	1
**F**	60–64	60–64	1	INFb-1a	3	B	INFb-1a	14	B	GA	40							1	0
**F**	15–19	15–19	1	INFb-1a	31	B	DMF	34	D									1	1
**F**	20–24	15–19	2	INFb-1a	20	C	FNG	55	D	CLA	28							1	1
**M**	20–24	20–24	1	pINFb-1a	67													1	1
**F**	45–49	40–44	1	INFb-1a	26	B	DMF	17	B	TFM	24							1	1
**F**	30–34	30–34	2	INFb-1a	25	B	DMF	50	B									9	0
**F**	55–59	45–49	1	TFM	57													1	0
**F**	40–44	40–44	1	GA	7	B	INFb-1a	29	B	pINFb-1a	3	B	INFb-1a	60				1	0
**M**	35–39	25–29	1	INFb-1a	92													1	0
**M**	30–34	30–34	1	INFb-1a	14	A	FNG	70										1	0
**M**	40–44	35–39	2	GA	6	B	FNG	100										1	1
**M**	35–39	35–39	2	INFb-1a	108													1	1
**M**	30–34	30–34	2	TFM	76	B												9	0
**F**	40–44	40–44	2	TFM	59													1	1
**F**	30–34	25–29	2	TFM	54													1	0
**F**	30–34	30–34	1	DMF	35	A	CLA	23										9	1
**F**	35–39	30–34	2	TFM	42													1	0
**F**	55–59	20–24	1															1	0
**M**	25–29	25–29	1	TFM	45													1	0
**F**	35–39	35–39	1	DMF	2	B	TFM	36										1	1
**M**	40–44	40–44	1	DMF	41													1	1

Sex: M = male; F = female. Age at first symptom and at diagnosis presented in full years. Disease activity assessed at diagnosis and classified according to Finnish guidelines as 0 = inactive; 1 = active; and 2 = very active. Reasons to switch: A = disease activity; B = side effects; C = disease activity; and side effects; D = other. Exposure to a DMT presented in months. Remission (0 = no; 1 = yes; 9 = cannot be assessed) as assessed at last follow-up visit and MRI (0 = no; 1 = yes) data available to support this. CLA, cladribine; DMF, dimethyl fumarate; DMT, Disease-Modifying Treatment; FNG, fingolimod; GA, glatiramer acetate; INF-1a, interferon 1a; NTZ, natalizumab; pINF-1a, pegylated interferon 1a; TFM, teriflunomide.
